# Supplementary material for: Development of an assay of plasma neurofilament light chain utilizing immunomagnetic reduction technology
Source: PLoS One. 2020 Jun 12;15(6):e0234519. doi: 10.1371/journal.pone.0234519 (PMC7292381; doi:10.1371/journal.pone.0234519)
Supplement: Attachment — Submitted filename: Response-R1.docx [file pone.0234519.s002.docx]

Responses to reviewers’ comments

| Comment | Response |
| --- | --- |
| Please ensure that your manuscript meets PLOS ONE's style requirements, including those for file naming. The PLOS ONE style templates can be found at http://www.plosone.org/attachments/PLOSOne_formatting_sample_main_body.pdf and http://www.plosone.org/attachments/PLOSOne_formatting_sample_title_authors_affiliations.pdf | The manuscript has been re-formatted according to the guidelines. |
| Please include your tables as part of your main manuscript and remove the individual files. Please note that supplementary tables (should remain/ be uploaded) as separate "supporting information" files | Tables have been integrated into the main body of the manuscript. |
| PLOS requires an ORCID iD for the corresponding author in Editorial Manager on papers submitted after December 6th, 2016. Please ensure that you have an ORCID iD and that it is validated in Editorial Manager. To do this, go to ‘Update my Information’ (in the upper left-hand corner of the main menu), and click on the Fetch/Validate link next to the ORCID field. This will take you to the ORCID site and allow you to create a new iD or authenticate a pre-existing iD in Editorial Manager. Please see the following video for instructions on linking an ORCID iD to your Editorial Manager account: https://www.youtube.com/watch?v=_xcclfuvtxQ | The corresponding author, Shieh-Yueh Yang, has an ORCID ID: 0000-0002-7850-4597. |
| Please provide an amended Funding Statement declaring this commercial affiliation, as well as a statement regarding the Role of Funders in your study. If the funding organization did not play a role in the study design, data collection and analysis, decision to publish, or preparation of the manuscript and only provided financial support in the form of authors' salaries and/or research materials, please review your statements relating to the author contributions, and ensure you have specifically and accurately indicated the role(s) that these authors had in your study. You can update author roles in the Author Contributions section of the online submission form. | Done. |
| Please also include the following statement within your amended Funding Statement. “The funder provided support in the form of salaries for authors [insert relevant initials], but did not have any additional role in the study design, data collection and analysis, decision to publish, or preparation of the manuscript. The specific roles of these authors are articulated in the ‘author contributions’ section.” | Done. |
| Please also provide an updated Competing Interests Statement declaring this commercial affiliation along with any other relevant declarations relating to employment, consultancy, patents, products in development, or marketed products, etc. | “MagQu Co., Ltd. and MagQu LLC do not alter our adherence to PLOS ONE policies on sharing data and materials.” is added into the revised manuscript. |
| Please include both an updated Funding Statement and Competing Interests Statement in your cover letter. | Done. |
| The authors give a brief description of the statistical analyses that were employed, but they did not describe the analyses used to ascertain the diagnostic accuracy of their NFL assay. They report sensitivity, specificity, and AUC values, but it is unclear what statistical tests were used to derive these values. | We thank reviewers for your comments. We have re-written the statistics subsection in the revised manuscript.  In addition, the following sentences are added in the statistics subsection for the tests of sensitivity, specificity and AUC:  “To discriminate PD/PDD (or AD) patients from NCs, receiver operating characteristic (ROC) curve analysis was conducted. The optimal cutoff of NFL concentrations was determined by the Youden index. The confidence interval of area under the curve, sensitivity and specificity was calculated using the DeLong’s nonparametric method.” |
| The authors also state that t-tests were used to compare continuous variables, but it is unclear what comparisons they are referring to. This statement is very vague as it is unclear if the authors are referring to paired t-tests for the reproducibility studies or whether they used a series a two-sample t-tests for the clinical group comparisons. If two-sample t-tests were used for the clinical group comparisons, this would be an incorrect approach. When comparing three or more groups, a one-way ANOVA should be used so that the groups are compared simultaneously and so that the p-values of groupwise comparisons that are statistically significant are adjusted for multiple comparisons. | The following sentences are added in the statistics subsection in the revised manuscript to clarify we were only interested in comparing normal control to disease groups and PD/PDD to AD groups:  “The data of NFL concentrations were checked for normality using the Kolmogorov-Smirnov test and the result showed suggested a violation in the normality assumption (*p* = 0.033; data not shown). Therefore, the comparison of NFL concentrations between groups (i.e., normal control vs. disease groups; PD/PDD vs. AD groups) was made using the Mann-Whitney U-test.“  The demographic information about Patient, including PD/PDD and AD, is added in Table 10 in the revised manuscript. |
| Did the authors check to ensure that the distribution of NFL values met the normality assumption? If they are not normally distributed, then non-parametric tests should be used to test group differences. | We found the NFL values violate the normality assumption. Therefore, we used Mann-Whitney U-test when comparing groups. The following sentences to illustrate this point are added in Statistics subsection:  “The data of NFL concentrations were checked for normality using the Kolmogorov-Smirnov test and the result showed suggested a violation in the normality assumption (*P* = 0.033; data not shown). Therefore, the comparison of NFL concentrations between groups (i.e., normal control vs. disease groups; PD/PDD vs. AD groups) was made using the Mann-Whitney U-test.“  Meanwhile, the Figure 3A is also revised. |
| Why did the authors report the diagnostic accuracy results in the Discussion section? This information should be included in the “Plasma NFL concentrations in patients with dementia” section. In addition, I emphasize the need to describe the statistical methods that were used to derive the AUC values, sensitivity, and specificity. Were these from a logistic regression model or a specific ROC function. Which of the stated statistical software packages were used for these analyses? | The diagnostic accuracy results have been moved to “Plasma NFL concentrations in patients with dementia”. The detailed information about statistics is described in Statistics subsection in the revised manuscript. |
| For AUC, sensitivity, and specificity, please report 95% confidence intervals with these values. | The values with 95% CI are added in Table 11. |
| Although clear differences in plasma NFL concentrations were observed among NCs and PD and AD patients, these diagnostic cut-off values should be validated in another group of more patients in further studies. | Thank for the valuable suggestion. We will do the validation by enrolling independent cohorts in the near future. |
